# Visualisation of variable binding pockets on protein surfaces by probabilistic analysis of related structure sets

**DOI:** 10.1186/1471-2105-13-39

**Published:** 2012-03-14

**Authors:** Paul Ashford, David S Moss, Alexander Alex, Siew K Yeap, Alice Povia, Irene Nobeli, Mark A Williams

**Affiliations:** 1 Institute of Structural and Molecular Biology, Department of Biological Sciences, Birkbeck, University of London, Malet Street, London WC1E 7HX, UK; 2 Pfizer Global Research and Development, Ramsgate Road, Sandwich CT13 9NJ, UK

## Abstract

**Background:**

Protein structures provide a valuable resource for rational drug design. For a protein with no known ligand, computational tools can predict surface pockets that are of suitable size and shape to accommodate a complementary small-molecule drug. However, pocket prediction against single static structures may miss features of pockets that arise from proteins' dynamic behaviour. In particular, ligand-binding conformations can be observed as transiently populated states of the *apo *protein, so it is possible to gain insight into ligand-bound forms by considering conformational variation in *apo *proteins. This variation can be explored by considering sets of related structures: computationally generated conformers, solution NMR ensembles, multiple crystal structures, homologues or homology models. It is non-trivial to compare pockets, either from different programs or across sets of structures. For a single structure, difficulties arise in defining particular pocket's boundaries. For a set of conformationally distinct structures the challenge is how to make reasonable comparisons between them given that a perfect structural alignment is not possible.

**Results:**

We have developed a computational method, Provar, that provides a consistent representation of predicted binding pockets across sets of related protein structures. The outputs are probabilities that each atom or residue of the protein borders a predicted pocket. These probabilities can be readily visualised on a protein using existing molecular graphics software. We show how Provar simplifies comparison of the outputs of different pocket prediction algorithms, of pockets across multiple simulated conformations and between homologous structures. We demonstrate the benefits of use of multiple structures for protein-ligand and protein-protein interface analysis on a set of complexes and consider three case studies in detail: i) analysis of a kinase superfamily highlights the conserved occurrence of surface pockets at the active and regulatory sites; ii) a simulated ensemble of unliganded Bcl2 structures reveals extensions of a known ligand-binding pocket not apparent in the *apo *crystal structure; iii) visualisations of interleukin-2 and its homologues highlight conserved pockets at the known receptor interfaces and regions whose conformation is known to change on inhibitor binding.

**Conclusions:**

Through post-processing of the output of a variety of pocket prediction software, Provar provides a flexible approach to the analysis and visualization of the persistence or variability of pockets in sets of related protein structures.

## Background

The availability of a protein's 3D structure may provide insight into its mechanism and a basis for rational design of small molecule modulators of its function. Key to understanding and modifying function in many proteins is knowledge of the structure of potential binding sites. Rational drug design strategies may then be used to design small molecules that bind to complementary features of such sites. In the absence of a structure containing a ligand, computational tools allow prediction of small molecule binding sites by scanning the protein's surface for pockets. These pockets must at least be of a size and shape that allows a ligand to bind with suitable specificity and affinity. Existing computational tools use a variety of methods to identify pockets, the simplest are based on local geometry and include PASS [1], LIGSITE [2], Pocket [3], PocketPicker [4], SURFNET [5], CAST [6] and fpocket [7]. Additional properties can be employed in pocket prediction, for example, LIGSITE-csc [8] and Concavity [9] combine structural information with sequence conservation scores, and Q-Site Finder [10] considers the energy of binding of hydrophobic probes to the protein's surface. It has been shown that the application of these tools to the analysis of individual static structures is useful in identifying a primary binding site [11,12], such as an enzyme's active site. However, proteins in solution are dynamic entities that explore conformational space over time due to side chain motions, local backbone flexibility and larger sub-domain or domain motions [13]. It follows that predictions of pockets based on single static structures may fail to detect potential binding sites, or features of such sites, that result from changes in their shape and size over time. The conformational selection hypothesis posits that bound conformations of proteins are often observed as transiently populated, high free-energy conformations of the *apo *protein. Ligand binding simply lowers the free-energy of the binding-capable conformation, thus increasing the probability and population of this state [14]. It is thus supposed that some points of the conformational space dynamically explored by the *apo *protein correspond to the pocket conformation of a ligand-bound form. Therefore, we can expect to gain insight into binding-capable pockets from inspecting conformational variants of proteins. Sets of variants can be derived from several sources: simulated ensembles created using Molecular Dynamics (MD) [15,16], Essential Dynamics (ED) [17], Normal Mode Analysis (NMA) [18] or constraint-based methods such as CONCOORD [19] and tCONCOORD [20]; solution-NMR conformational ensembles; multiple structures of the same protein solved in different crystal forms, or with different ligands or experimental conditions. It has also been shown that the structure-space explored within sets of homologues correlates with that observed with MD simulations [21], consequently homologous superfamilies of proteins provide other potentially useful sets of variant structures.

### Difficulties comparing predicted pockets between different programs or across related structures

**Figure 1:**
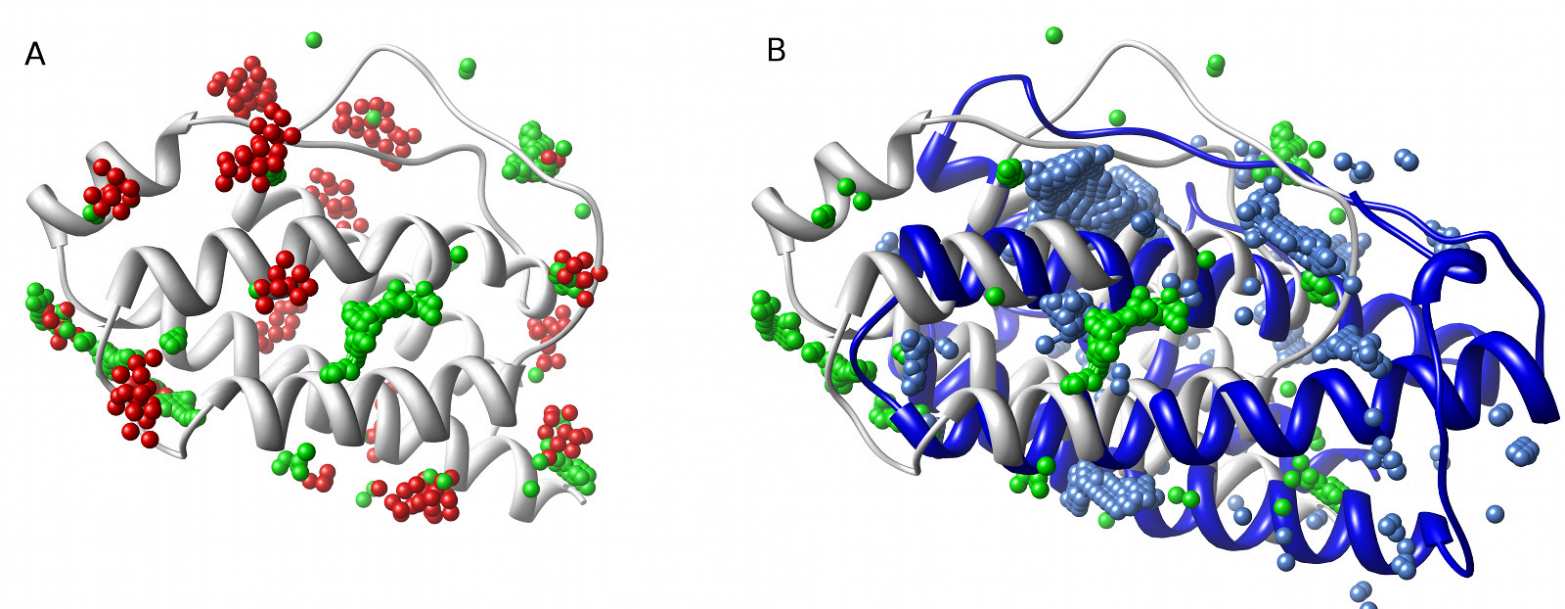
**The difficulties of global comparison of pockets on protein surfaces**. **(A) **Pocket predictions from PASS (red) and LIGSITE^-cs ^(green) for the same interleukin-2 (IL-2) structure (ribbon, PDB:1M47). The similarities and differences between these predictions are readily visualized, but how can identification of common features be automated? **(B) **Homologous structures: The surface pockets of IL-2 (white ribbon, green spheres) [PDB:1M47] and its distant homologue leukaemia inhibitory factor (LIF) (blue ribbon, blue spheres) [PDB:1LKI], both represented by LIGSITE^-cs ^spheres, show little overlap. Is this because the pockets are genuinely unrelated, or because of the difficulty of making a 'correct' structural alignment?.

**Figure 2:**
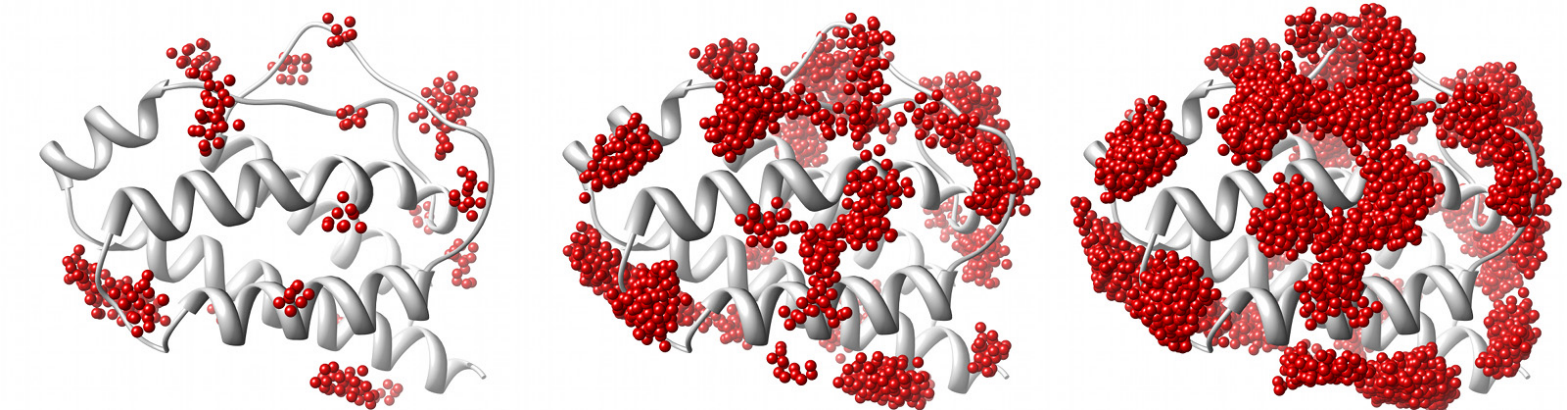
**The difficulty of comparing pocket predictions for many conformers of a protein**. PASS (red) pocket predictions for an increasing number of conformers are superimposed onto a ribbon structure of human IL-2 [PDB:1M47]. From left to right, the results for 1, then 10 and 50 superimposed conformations. There is a greater spread of predicted pocket locations as the number of conformers increases. However, in this representation, the spread arises from both local structural variation of pockets and the different reorientation of each conformer required for the best global alignment.

Given a set of related protein structures, how do we compare their pockets and the variation within the set? An approach is to designate particular pockets, 'Pocket A', 'Pocket B', etc., and in each case perform some detailed analysis of the pocket's geometry and other characteristics. This can be effective with a single, highly-conserved pocket, such as an enzyme's active site [22]. However, a problem is that it is not clear how to consistently and unambiguously define each pocket in terms of its boundaries in cases where there are many possible sites i.e. are neighbouring pockets best considered as two distinct entities, or as part of a single contiguous whole (for discussion see [23,24])? An additional complication is that for any given single structure we can obtain different predicted pockets depending on which software we choose to run. A simple case of two different programs' (PASS and LIGSITE) outputs for a single structure of human interleukin-2 (IL-2) is illustrated in Figure 1A, which shows only partial overlap of the clusters of pocket points for the two programs. In comparing pockets predicted in homologues, the issue is to identify whether apparent differences in pocket locations are simply a result of problems with structural alignment (Figure 1B). The visual comparison of pockets in diverse conformations of a single protein can also be affected by difficulties with alignment. Figure 2 illustrates how the spread of PASS predicted pocket points increases with the number of conformations used, an effect caused both by local structural variations and differences in global orientation required to produce the best overall alignments of the structures.

Analysing a large set of conformers may help identify structural variation in otherwise persistent pockets, and transient pockets that are observed in only some members of the set. Finding evidence of variable or transient pockets may suggest novel targets and provide a useful adjunct to rational drug design strategies. Recent analyses have addressed the evidence for transient pockets by applying PASS to snapshots of MD trajectories of a member of the B-cell lymphoma family (Bcl-X*_L_*), human IL-2 and mouse double minute 2 (MDM-2) [25] and identifying pockets across snapshots by clustering on pocket volume and the overlapping pocket-lining residues, finding several distinct transient pockets in these systems. In a follow-up study, tCONCOORD produced comparable pockets to the MD simulations for Bcl-X*L *and MDM-2 [26]. Another recent approach uses the fpocket prediction algorithm, with a specific front-end for analysis of MD ensembles and trajectories (MDpocket [27]), to understand how pockets change across many different conformations by mapping pocket predictions for each structure onto a fixed spatial grid, calculating the frequency of occurrence of a pocket at each grid point, and visualising this as a density map superimposed on a reference protein structure [7]. Here we propose a similarly probabilistic, but otherwise distinct, approach that differs in utilising the output of any suitable geometric pocket prediction program, including PASS, LIGSITE, fpocket and SiteMap [28], and maps each set of predictions to its corresponding protein structure before calculating overall probability densities. We present a method that provides standardised visual analyses of predicted pocket variation across any suitable set of related protein structures. The approach of first mapping the location of pockets to the protein's atoms and residues removes the art factual influence of differences in global orientation of structurally aligned sets and provides for weighting of probability densities by residue conservation. Consequently, the method is particularly advantageous in analyses of sets of conformers or homologues that differ markedly in structure.

## Algorithm

**Figure 3:**
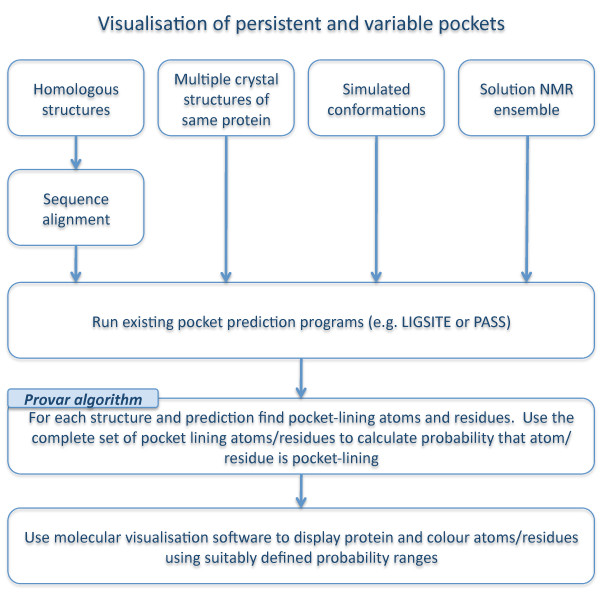
**Workflow for visualisation of pockets in ensembles of structures using Provar tools**.

We have developed a method that gives visual insight into the variability of predicted pockets whilst overcoming the difficulties outlined above. A flowchart outlining the potential utilization of the method to analyse/identify regions of interest using multiple pocket predictions is given in Figure 3.

**Figure 4:**
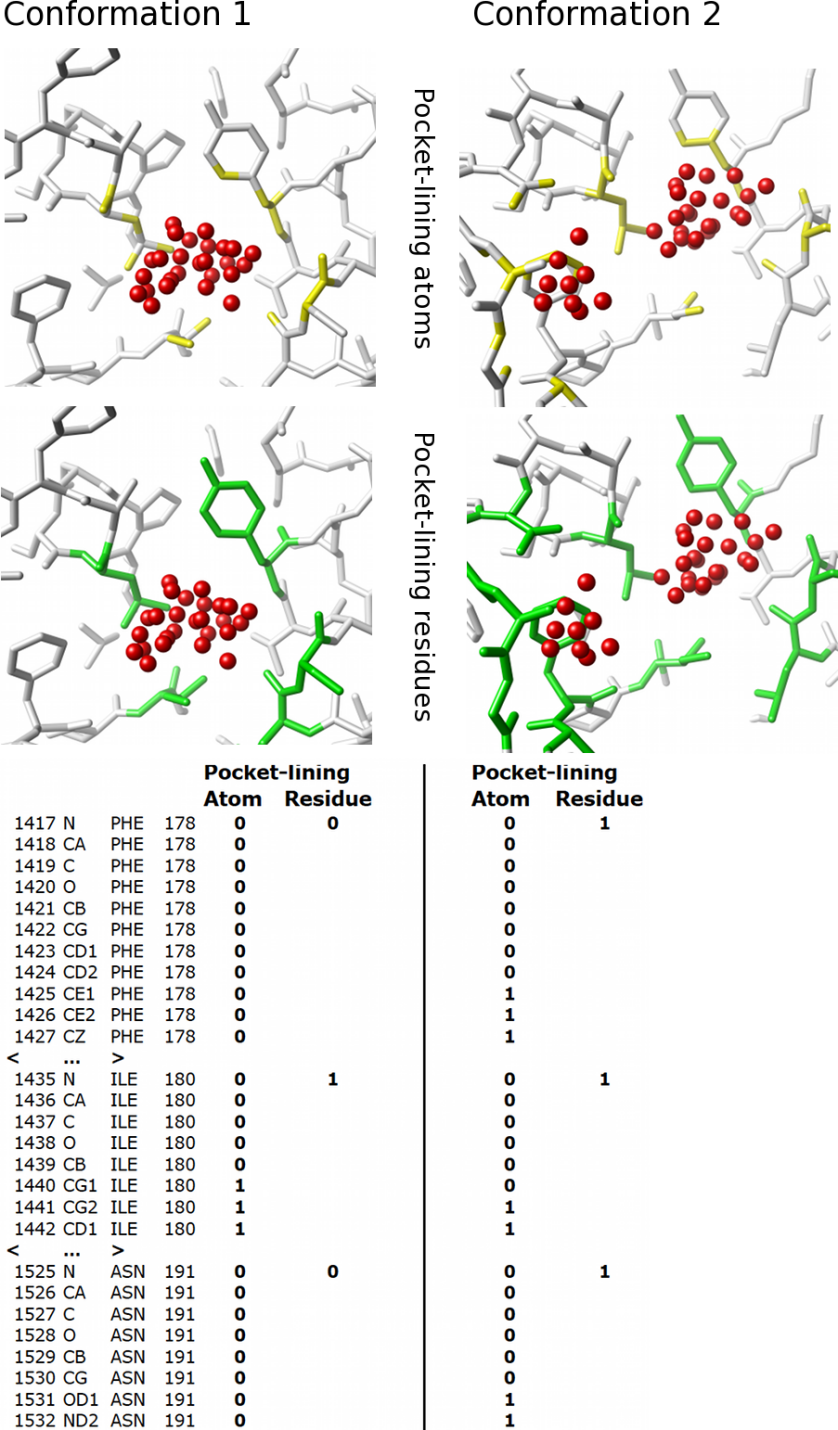
**Provar algorithm scores pocket-lining atoms and residues on each structure**. Illustration of Provar applied to the same pocket on two simulated conformations of Abl kinase [PDB:2GQG]. For each structure, pocket atoms (top panels, yellow) are scored as pocket-lining if sites predicted to be pocket-lining (red spheres) are within a defined cut-off distance. Residues that contain pocket-lining atoms are also pocket-lining (mid panels, green). For each structure this generates one set of scores for each structure's atoms and another for its residues. The scores of individual structures can then be readily combined to calculate the proportion of the equivalent atoms or residues being pocket-lining within a set of structures.

At the heart of the approach is an algorithm, Provar (Probability of variation), for automatically identifying and scoring pocket-lining residues or atoms, which is outlined in Figure 4. For each set of *n *related structures *{s_1_,s_2_,s_i_,...,s_n_}*, we also have *n *pocket predictions *{d_1_,d_2_,d_i_,...,d_n_} *from programs such as PASS or LIGSITE whose predictions are collections of points in space. Together we have a set of pairs of structures and predictions: *{{s_1_,d_1_},{s_2_,d_2_},{s_i_,d_i_}...,{s_n_,d_n_}}*. For each of these matched pairs (*i*), Provar determines that an atom (*k*) of a structure is pocket-lining if it lies within a parameter-defined cut-off distance of any pocket prediction point and gives it a score of 1 (*a*_*k *_= 1). For each amino acid (*j*) in the sequence, should *a*_*k *_= 1 for any of its atoms, the amino acid is given a score of 1 (*r_j _*= 1). This process results in an array of atom and residue values for each structure prediction pair: *{s_i_,d_i_} *→ *{a*_1_,*a*_2_,*a_k_,..*.,*a_l_}_i _*where *a*_*k *_∈ *{*0, 1*} *and *l *is the number of atoms in the protein and *{s_i_,d_i_} *→ *{r*_1_,*r*_2_,*r_j_,..*.,*r_m_}_i _*where *r_j _*∈*{*0, 1*} *and *m *is the number of residues in the sequence.

If we are dealing with sets of structure-prediction pairs containing the same number of atoms and residues (e.g. CONCOORD conformers, or NMR ensembles) we can now assign an atom-level probability score for the *k*th atom:

(1) $$p_{k}=\frac{1}{n}\overset{n}{\underset{i=1}{\sum }}a_{ik}$$

i.e. the proportion of structures in which the atom is pocket-lining. We can do a similar calculation for the *j*th residue:

(2) $$p_{j}=\frac{1}{n}\overset{n}{\underset{i=1}{\sum }}r_{ij}$$

If we have a set of homologous structures, then their sequences must first be aligned. The probability calculation in this case must take into account the number of structures *n_j _*that have an aligned residue at position *j*. Alignments are performed on sequences comprising those residues having coordinates in the PDB file, as this ensures missing residues don't bias the probability calculations.

(3) $$p_{j}=\frac{1}{n_{j}}\overset{n}{\underset{i=1}{\sum }}r_{ij}$$

It is difficult to define an atom-based equivalent to Equation 1for homologous structures as aligned residues may have different numbers of atoms. However, a residue-based average of atom scores can be defined as follows:

(4) $${\bar{p}}_{j}=\frac{1}{n_{j}}\overset{n}{\underset{i=1}{\sum }}\left(\frac{1}{l_{ij}}\overset{l_{ij}}{\underset{k=1}{\sum }}a_{ijk}\right)$$

where *l_ij _*is the number of atoms in the *j*th residue of the *i*th structure and *α_ijk _*is the atom-based score for the *k*th atom of the *j*th residue for the *i*th structure. This can be applied both to structures with the same sequence and to homologues to give the proportion of the atoms of each equivalent residue in the set of structures that are pocket-lining, and can usefully distinguish those residues which contribute most to the formation of the pocket.

The probability values for each residue can then be displayed on a protein structure in a number of ways. Our software writes the probability values as a percentage to the B-factor column of the PDB file of a user chosen representative structure from the set. This structure can then be rendered using any suitable molecular graphics program. We show how the Provar algorithm provides a practicable solution to the problems outlined in the Introduction when considering pocket predictions from multiple programs on a single structure, across homologous structures and within sets of generated conformations.

## Results

### Visual comparison of alternate pocket predictions, homologous structures and variation among multiple conformations

**Figure 5:**
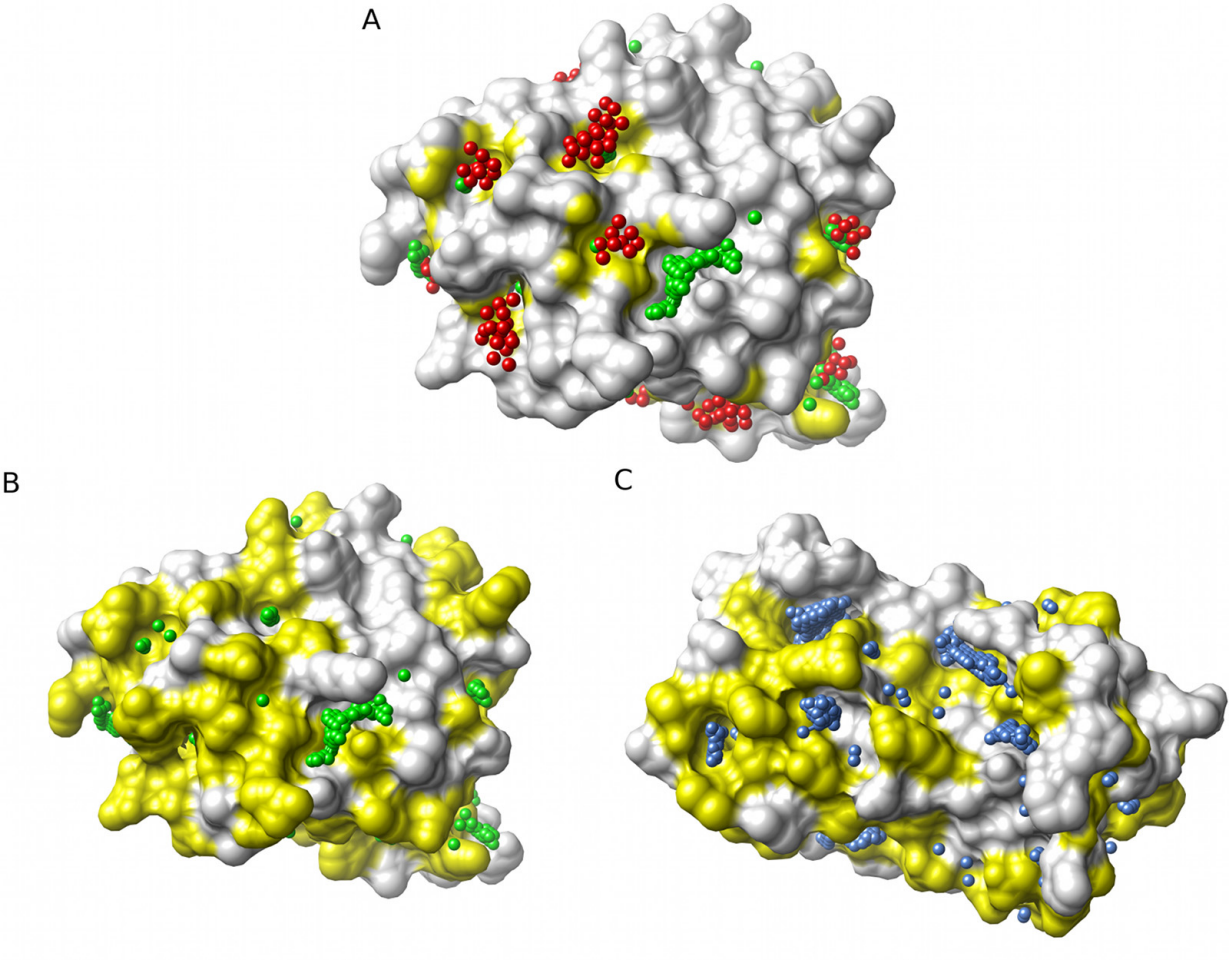
**Mapping pocket locations to the protein atoms and residues simplifies comparison between methods and proteins**. **(A) **Proteins of identical sequence can be compared at either residue or atomic levels. Combining the atomic scores derived from the PASS and LIGSITE^-cs ^results for IL-2 (Figure 1A) identifies common pocket-lining atoms (Provar score = 1), which can be mapped back to protein surface (yellow). **(B) **Homologous proteins can be compared at the residue level. Equivalent pocket-lining residues (yellow) of IL-2 and LIF, highlighted on a surface representation of IL-2, were identified as residues with a Provar score of 1 for LIGSITE^-cs^-derived sequence aligned residues (c.f. Figure 1B). **(C) **Residues with a score of 1 are mapped onto the surface of LIF.

Figure 5A illustrates the use of Provar to represent the PASS and LIGSITE predictions from Figure 1A on the surface of IL-2, with atoms colored yellow only if both programs mark an atom as pocket-lining (*p_k _*= 1). For the more difficult problem involving IL-2 and its distant homologue LIF (from Figure 1B) - we can now readily visualise surface patches of coincident pockets between the two homologues where yellow patches represent equivalent residues that are pocket-lining in both structures (*p_j _*= 1) mapped to the surface of either IL-2 (Figure 5B) or LIF (Figure 5C).

**Figure 6:**
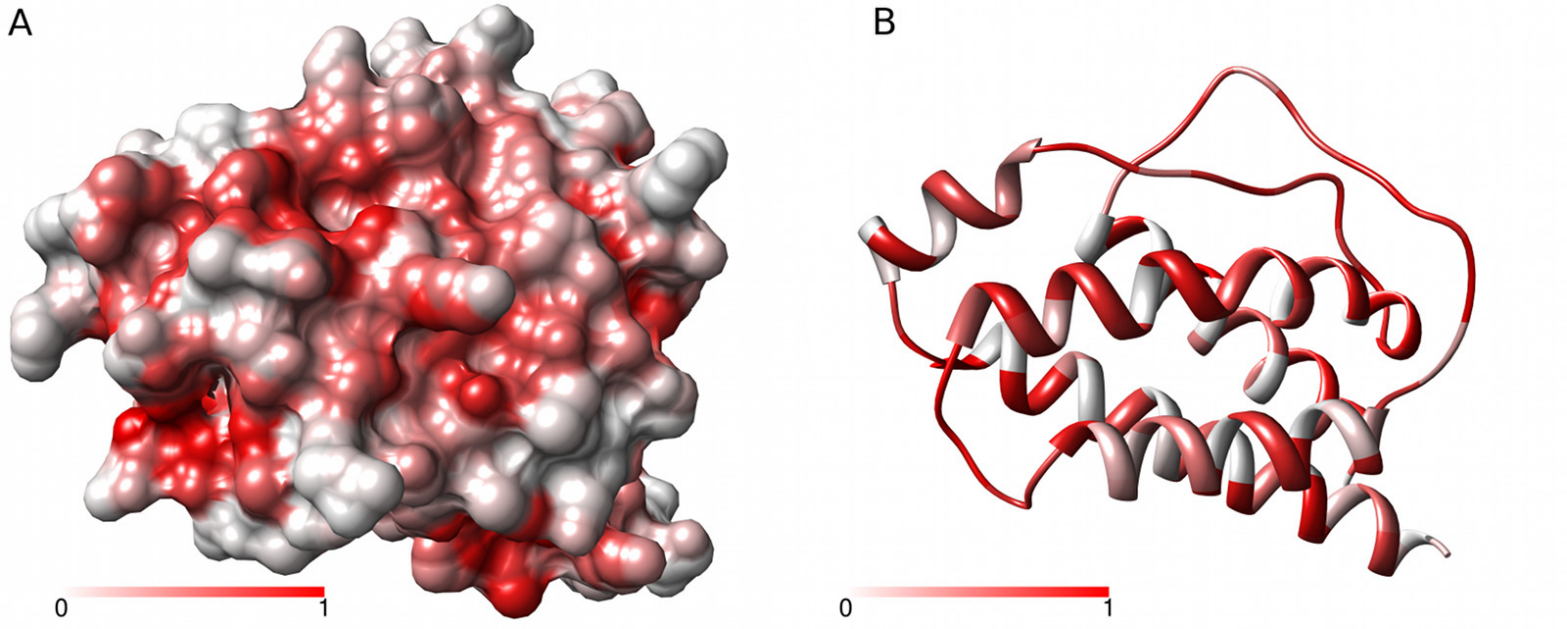
**Provar discriminates atoms and residues which persistently or variably contribute to pockets in an ensemble**. **(A) **The atomic Provar score for a set of 50 conformers of IL-2 generated with tCONCOORD readily distinguishes those atoms persistently involved in pocket formation (dark red) from those only involved in pocket formation in minority of structures (light red). **(B) **Equivalent residue scores on a ribbon representation.

Figure 6 provides the Provar solutions to the problem posed by multiple generated conformations of IL-2 (from Figure 2). The probability that an atom is pocket-lining across all 50 structures is indicated on a continuous scale on the surface (Figure 6A), while the residue-level calculation using Equation 2 is applied to a ribbon representation (Figure 6B). This provides a simple way of identifying the atoms/residues involved in the most persistent pockets (darker reds) and regions that harbour variable pockets (lighter reds).

### Visualising the most conserved pocket-lining residues across a kinase superfamily

**Figure 7:**
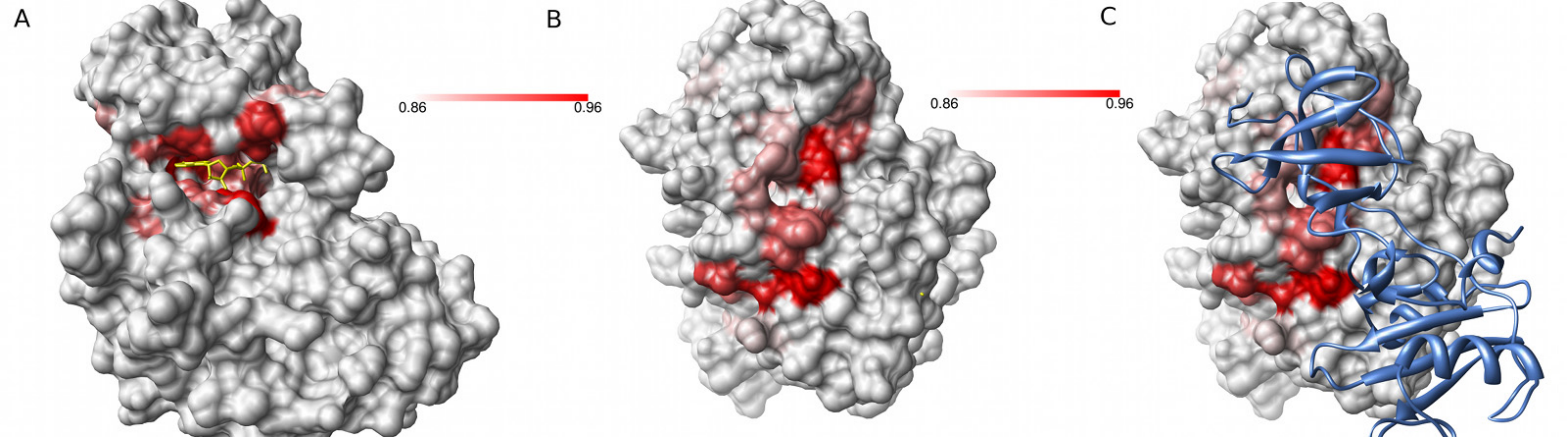
**Mapping the residues of a kinase superfamily that most frequently form pockets highlights two regions**. A Provar analysis of 93 members of the phosphorylase/kinase superfamily mapped onto a representative structure [PDB:2R3I]. **(A) **Residues which are very frequently pocket-lining (light red - 86% of structures - to dark red - 96%) cluster around the active site region. The active site is indicated by ATP in yellow, superimposed from an ATP-bound structure [PDB:2PVF]. **(B) **A second region in which residues are very frequently involved in pocket formation is found on the opposite side of the kinase from the active-site. **(C) **Some of the conserved features highlighted in **(B) **may be important in mediating protein-protein interactions. For example, it is known that one member of this superfamily, Abl kinase, is regulated via an interaction at this site with its own SH3 domain (superimposed SH3 domain from [PDB:2FO0] blue ribbon).

Protein kinases form a large and well conserved superfamily that are of particular interest in drug discovery. For example, constituent activity of Abl kinase resulting from the Bcr-Abl gene fusion leads to chronic myeloid leukaemia (CML) [29]. Specific small molecule inhibitors of the Abl kinase active site have been developed and approved as therapy for CML. Using Provar we can conveniently summarise pocket-lining residue conservation across all superfamily members onto a single structure to highlight regions that show conservation of predicted pockets (Figure 7). As expected, residues around the active site (indicated with superimposed ATP) are clearly highlighted (Figure 7A) due to conservation of structure and function. Another distinct region is found on the other side of the protein (Figure 7B) and the high conservation of this pocket is likely to have functional relevance across the superfamily. For the specific case of Abl kinase, this region is known to form part of the interface of the auto inhibitory interaction with its own SH3 domain, and this fact is suggestive of a conserved role for this pocket in mediating protein-protein interactions. In this case, the red colouration indicates that the residue in that alignment position is pocket-lining in most or all homologues. There may, of course, be considerable variability both in the actual residue present and the orientation of its side-chain that gives scope for binding other proteins or, in a drug-design context, a small molecule ligand with suitable specificity for a particular kinase.

### Visualisation of pocket-lining atoms from a simulated ensemble of Bcl-2 conformers

**Figure 8:**
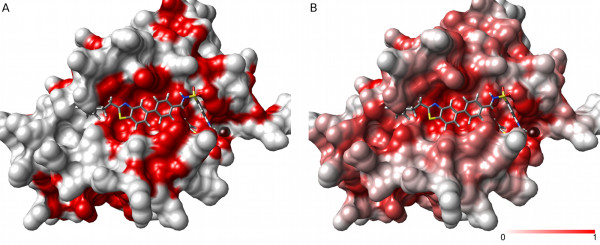
**Scoring a simulated ensemble of apo structures of Bcl-2 identifies variation in a known ligand binding site**. **(A) **Pocket-lining atoms (red) identified from a PASS analysis of the *apo *crystal structure of Bcl-2 [PDB:1GJH] capture only part of the binding site of an acyl-sulfonamide-based ligand, here illustrated by superimposing the ligand from [PDB:2O1Y]. **(B) **Provar analysis of a tCONCOORD-generated ensemble of *apo *structures indicates that pocket-lining residues (red) are found along the full-length of the binding groove in most structures.

Bcl-2 is part of a family of apoptosis regulators that can homo-dimerize or hetero-dimerize with other members of the family to form pro- or anti-apoptotic complexes. As pro-apoptotic proteins such as BAK and BAD can be inhibited by the binding of Bcl-2 (or its homologue Bcl-X*_L_*), specific inhibitors of these protein-protein interactions are of interest in oncology research [30]. In Figure 8, we compare PASS pocket predictions for the *apo *crystal structure of Bcl-2 with a Provar analysis of 250 tCONCOORD generated conformations. For the crystallographic Bcl-2 structure (Figure 8A), pocket predictions, in red, coincide with a large portion of the known protein-protein interface groove. Small molecule drugs have also been found to target this interface in both Bcl-2 and Bcl-X*_L _*and superposition of an acyl-sulfonamide-based ligand from a *holo *structure shows how it follows the interface groove, but extends outside the pocket identified by PASS. In contrast, Provar analysis of the tCONCOORD ensemble shows that an extension to the pocket is found at the left in a substantial proportion of conformers (Figure 8B). In the *apo *crystal structure, the pocket is bounded at the left by Glu-136 (with which the superimposed ligand is seen to clash in Figure 8), this residue reorients in other conformers (and the actual *holo *structure) opening up a larger groove for binding. Although, the members of the *apo *tCONCOORD ensemble do not fully recapitulate the Bcl-2 *holo *conformation, Provar analysis of the ensemble shows that 87% of the atoms in the binding site of Bcl-2 for this inhibitor are identified by PASS to be pocket-lining in at least 25% of the structures (as opposed to 49% of these atoms in the *apo *crystal structure). Additionally, away from the main groove, the probability map indicates where atoms flagged as pocket-lining in the single structure turn out to be less important across the ensemble. For example, prominent pocket-lining atoms at the very top of the crystal structure in Figure 8A are found to have much lower probability in the ensemble than the main binding groove (Figure 8B).

### Application to simulated ensembles relevant to inhibition of protein-protein interactions

It is of interest to more broadly investigate the behaviour of protein-protein interfaces (PPIs) known to bind small-molecule inhibitors. Whereas protein-protein interfaces are generally rather flat and lacking pocket features, it has been observed in number of cases that pockets are stabilised in the presence of a small-molecule, which consequently acts to inhibit protein-protein complex formation [31]. Are these pockets discernable in the *apo *proteins in the absence of inhibitor? Is it common that a substantial proportion of the inhibitor binding sites seen in the complexes comprise variable pockets in the *apo *state and that variable features can be recovered through tCONCOORD simulation of the *apo *structure accompanied by Provar scoring of pockets?

We have investigated all 11 proteins in the 2P2I database of protein-protein interface inhibitors [31] that have been structurally characterised. The results of pocket analyses of the crystal structures and tCONCOORD ensembles of the *apo *form of these proteins - in respect of the pocket-lining character of the atoms known to interact with a small molecule ligand - are given in Table 1. Here we see that on average LIGSITE^-cs ^identifies almost half of the known binding-site atoms as pocket-lining in the *apo *crystal structures, this proportion falls to somewhat less than one-third that are persistently pocket lining in the dynamic ensemble (we define persistent as occurring in more than75% of conformations), but rises to an average of 72% of the binding-site atoms that are found to be pocket lining in at least 25% of the ensemble. These trends are mirrored by results obtained for PASS and fpocket analysis of the same structures. It is also clear, from Table 1 that the precise results of these ensemble-based pocket analyses are rather different for each program, with PASS and fpocket identifying, on average, successively fewer binding-site atoms as pocket-lining. This order is not preserved for every structure, and whether or not pockets are found, presumably, depends upon precise geometric features of pockets in individual proteins to which each algorithm is differently sensitive. Although, on average, LIGSITE^-cs ^recovers more of the known binding sites, this is substantially due to it identifying more of the protein as a whole as pocket-lining. According to LIGSITE^-cs^, 60% of all the atoms averaged over all the proteins are pocket-lining in more than 25% of conformations. This is due to there being many small pockets in the ensemble to which LIGSITE^-cs ^is sensitive (using the default parameters). Both PASS (41%) and fpocket (32%) are more conservative in predicting pockets across the protein surface and, although they on average identify fewer of the binding sites' atoms as pocket, are slightly more specific. Despite these variations, it is the case that all the prediction programs identify substantially more binding-site atoms as persistently or variably pocket-lining than in the protein as a whole. This is also true for the majority of analyses of individual proteins i.e. that the distribution of Provar scores of binding site atoms is significantly different from the protein as a whole and biased toward a higher probability of being identified as pocket-lining in the ensemble. Overall, these results support the notion that transient or variable features of pockets are at least a majority feature of protein-protein inhibitor binding sites.

**Table 1:** Ensemble analysis of the binding sites of protein-protein interaction inhibitors

Protein	Binding site size (atoms)	Method	Percentage of site identified as pocket-lining		
			*apo *structure	**Provar Score **> 0.75	**Provar Score **> 0.25
		PASS	38	21	64
Bcl-X*_L_*	56	LIGSITE	73	69	91
		fpocket	52	36	89
		PASS	46	15	*50*

MDM2	48	LIGSITE	52	38	77
		fpocket	38	6	*45*
		PASS	29	26	69

XiapBir3	42	LIGSITE	67	52	98
		fpocket	31	24	83
		PASS	25	16	*54*

XiapBir3	63	LIGSITE	57	44	87
		fpocket	30	19	74
		PASS	70	35	75

ZipA	20	LIGSITE	35	5	*60*
		fpocket	0	15	*15*
		PASS	46	23	54

HPVE2	97	LIGSITE	44	26	56
		fpocket	33	2	53
		PASS	38	7	58

IL2	60	LIGSITE	22	22	62
		fpocket	0	2	*2*
		PASS	53	53	68

HIV-1 Integrase	19	LIGSITE	37	16	*47*
		fpocket	68	5	*58*
		PASS	6	0	*29*

TNFa	34	LIGSITE	3	0	*44*
		fpocket	0	0	*3*
		PASS	0	0	*69*

TNFR1a	16	LIGSITE	50	38	100
		fpocket	0	0	69
		PASS	62	30	57

MDM4	81	LIGSITE	44	31	68
		fpocket	38	0	42
		PASS	38.5	20.5	59

**Mean Values**	49	LIGSITE	49	31	72
		fpocket	37.5	8.5	48

Provar analysis of a tCONCOORD generated ensemble of 250 structures of the *apo *form of each of the 11 proteins in the 2P2I database identifies a greater proportion of the known binding site atoms as pocket-lining than analysis of the crystallographic structure alone. The proportion of inhibitor binding-site atoms found to be persistently or variably lining pockets (defined as pocket-lining in at least 25% of conformers) is significantly greater than for the protein as a whole in 21/33cases. (An italic number in the final column indicates the minority of cases in which the proportion is not significant at the *p *< 0.05 level). Further details of the structures are given in Table 4.

Considering these same ensembles of *apo *structures from the perspective of the protein-protein interface, there are proportionately fewer pocket-lining atoms in such interfaces, although still greater than the protein surface as a whole. On average both the binding-site and protein-protein interface have a greater proportion of atoms that are variably pocket lining according to PASS (38.5% binding-site vs. 35.5% PPI vs. 27% protein surface) and fpocket (39.5%,36%,32%). This dynamic pocket formation is in contrast to earlier analysis of the *apo *crystal structure dataset [31] using Q-Site Finder which found relatively few "static" pockets at these interfaces. Given the large numbers of atoms in total in this set of interfaces, the average differences found here are significant, but it is also the case that excess pockets in PPIs are only found in half of the individual analyses (Table 2). Consequently, there is no strong evidence on which to make general statements about the relative variability of drug gable PPI interfaces in terms of pocket formation. Clearly all these interfaces can form pockets able to bind small molecule ligands, but it seems more likely that this is a very local feature of the protein-protein interface rather than a consequence of an average property of it.

**Table 2:** Ensemble analysis of pockets at protein-protein interfaces

Complex	Protein-protein interface size (atoms)	Method	Percentage of interface identified as pocket		
			*apo *structure	**Provar Score **> 0.75	**Provar score **> 0.25
Bcl-X*_L_*		PASS	28	16	*42*
+	74	LIGSITE	66	39	83
Bak		fpocket	41	20	50

MDM2		PASS	27	11	*45*
+	193	LIGSITE	41	30	74
p53		fpocket	31	8	46

XiapBir3		PASS	30	9	*55*
+	128	LIGSITE	42	35	*83*
Caspase 9		fpocket	19	13	*59*

XiapBir3		PASS	17	0	*43*
+	42	LIGSITE	21	17	*81*
SMAD		fpocket	21	0	65

ZipA		PASS	54	13	65
+	63	LIGSITE	44	19	*57*
FtsZ		fpocket	10	2	*19*

HPVE2		PASS	42	22	51
+	109	LIGSITE	44	22	58
HPVE1		fpocket	17	3	32

IL2		PASS	30	5	55
+	103	LIGSITE	20	17	*57*
IL2-R		fpocket	4	1	*19*

Integrase		PASS	42	42	64
+	33	LIGSITE	45	18	*48*
LEDGF		fpocket	52	3	51

TNFa		PASS	23	9	*44*
trimer	189	LIGSITE	34	18	*63*
interface		fpocket	15	3	*31*

TNFR1a		PASS	24	5	*34*
+	41	LIGSITE	46	10	*71*
TNFb		fpocket	0	0	*34*

MDM4		PASS	55	24	48
+	71	LIGSITE	38	32	52
p53		fpocket	44	0	47

		PASS	34	14	49.5
**Mean Values**	95	LIGSITE	40.5	23.5	66
		fpocket	23	5	41

Provar analysis of a tCONCOORD generated ensemble of 250 structures of the *apo *form of each of the 11 first-named proteins in the complex. The proportion of protein interface atoms found to be persistently or variably lining pockets is, on average, significantly greater than for the protein as a whole. However, this enhanced variability is small and only found to be significant for 14/33 individual analyses. (Italic number in final column indicates the cases in which the proportion is notsignificantly greater at the *p *< 0.05 level).

### Visualisation of IL-2 homologues shows conserved pockets at the α, β and γ receptor interfaces

**Figure 9:**
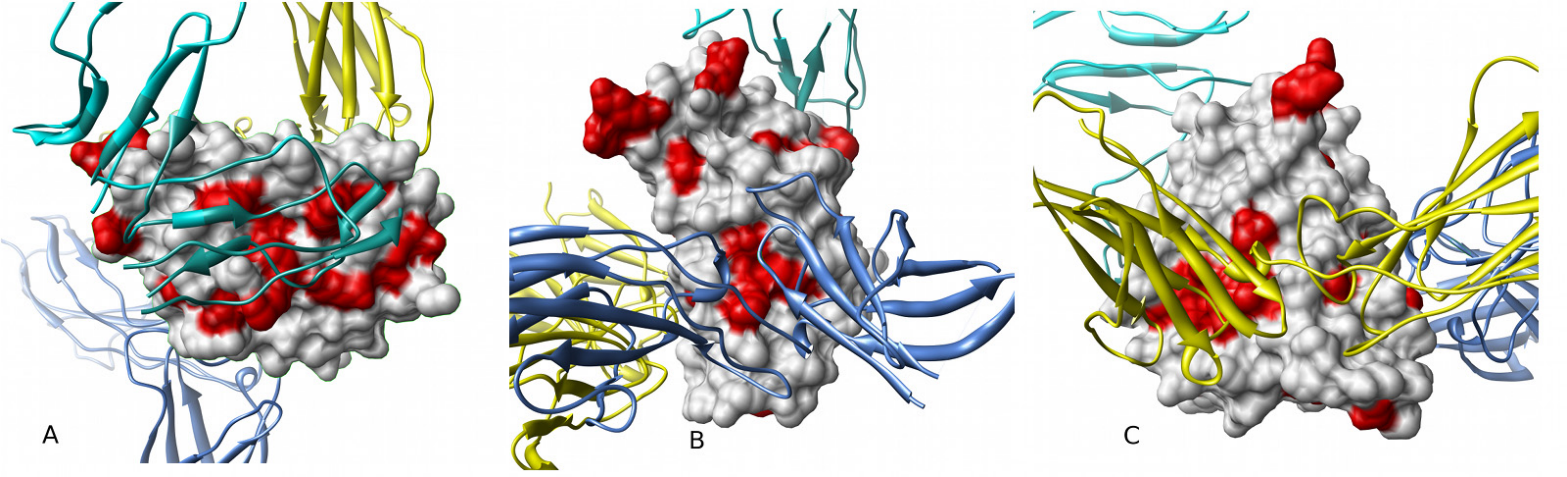
**Residues lining pockets across apo IL-2 superfamily members overlap with IL-2-receptor interfaces**. The residues most often involved in pocket formation (defined as those residues having the top 25% of Provar scores, ≥ 0.364 in this case) across 17 *apo *structures of functionally diverse IL-2 homologues are colored red on the molecular surface of IL-2 in a receptor bound conformation [PDB:2B5I]. These pocket forming residues, thus identified in a substantial subset of the *apo*-structures, overlap the IL-2:IL-2 receptor interfaces **(A**, **B**, **C) **with respectively the receptor *α*, *β *and *γ *chains, suggesting that the location of their receptor interaction sites is well conserved across the family despite low sequence identity.

The cytokine IL-2 binds the IL-2 receptor (IL-2R) at three distinct protein-protein interfaces with the receptor's *α,β *and *γ *chains [32]. We might anticipate that these receptor-binding interfaces will be used by other members of the superfamily to form complexes with their specific receptors. Provar's summary of the surface pockets found in the *apo *structures of this superfamily does indeed show a strong overlap between the most conserved pockets on these small and diverse proteins and the sites of interaction of the *α*, *β *and *γ *receptor chains found in the IL2-receptor complex (Figure 9). Although there is greater variation of the surface features of this superfamily homologous cytokines than in the case of kinases discussed above, the overlap of the IL2 binding interfaces and the relatively most conserved pockets is clearly discernable. If we consider analyses using each of PASS, LIGSITE^-cs ^and fpocket (Table 3), then we find that the most conserved pockets (defined by the pocket-lining residues having a Provar score in the highest quartile) recover only ~ 30-42% of the known protein-protein interfaces i.e. conserved pockets are a minority feature of these interfaces. Thus, the sensitivity of detection of the extent of protein-protein interfaces by detection of conserved pockets is low. However, we also find that pockets are very specifically conserved at these interfaces (specificity is ~ 71-82%) and that there are very few highly conserved pockets elsewhere on the protein surface. This high degree of specificity means that it is tempting to hypothesise that it may be possible to identify functional binding pockets in other systems through analysis of superfamily members even in the absence of structures showing the interactions with their receptors. The success of such an approach would, of course, depend on whether the recognition mechanism for the superfamily utilised pockets.

**Table 3:** Conserved pockets in the IL-2 superfamily at the known IL2 receptor interfaces

Pocket prediction (ensemble scoring)	Highly conserved pocket residues at interface	Sensitivity	Specificity
PASS (Provar)	13	30.2	81.8

Ligsite pockets (Provar)	13	30.2	80.5

fpocket (Provar)	18	37.2	71.4

fpocket (MDpocket)	21	48.8	46.8

There are three distinct interfaces made between IL-2 and the subunits of the IL-2 receptor, which together involve 43 residues of the 120 residue IL2 protein (with only 1 residue that contacts two subunits). Investigation of pockets in 17 *apo *structures from the highly diverse IL-2 superfamily (pair wise sequence identities of 7.5-13.3%) using the Provar scoring methodology (Equation 4) shows that there are several relatively highly conserved pocket-lining residues which lie at the receptor interfaces (Figure 9). This is suggestive that these pockets are functionally significant in a substantial proportion of the family. Selecting the 25% of residues with the highest Provar scores from analyses with PASS, LIGSITE^-cs ^and fpocket prediction methods shows a similar pattern of high specificity (TN/(TN + FP)), but low sensitivity (TP/(TP + FN)) for interface residues. Scoring fpocket predictions with an alternative ensemble approach (MDPocket) that uses spatial averaging, and is thus sensitive to superposition error, has high scores in similar locations, but also scores more areas of the protein surface similarly highly and thus has lower specificity.

The IL-2 superfamily data also provide an opportunity to contrast the ensemble averaging approach of Provar to that of fpocket/MDpocket. As described in the Algorithm section, both programs take a probabilistic approach to scoring pockets found in ensembles of structures. In the case of fpocket/MDpocket, structures are superimposed and the proportion of structures in which pockets are found at each point in space is determined. Spatially averaged pocket densities determined by the fpocket algorithm can be mapped by MDpocket to the neigbouring surface atoms of a reference structure. In contrast, Provar maps pockets to the local surface atoms for each structure in the ensemble and then accumulates these atomic scores over the ensemble. When the structures in the ensemble are fairly similar, the differences between these two approaches to averaging is small in terms of the final map of pockets to the protein surface. However, in a case such as the IL-2 superfamily, whose representative members have less than 15% pair wise sequence identity and rather diverse structures that are difficult to superimpose, pockets found on one structure's surface may be quite far from the surface of the reference structure. Thus any final mapping of spatial densities may be spread across the surface in an art factual way. The consequence of this lack of direct association with a local surface is that, in the IL2 superfamily case, fpocket/MDpocket ensemble averaging scores more of surface residues as pocket-lining, and that consequently more of the interface residues are associated with a high score. However, specificity is much lower than fpocket/Provar as more non-interface sites are also considered conserved. Of course, this case is particularly difficult for fpocket/MDpocket, but the lesson is that one or both approaches may be useful depending on the structure set in question.

### Highlighting variation in the contribution of residues to pocket formation among homologues and simulations of apo IL-2 also highlights regions known to undergo conformational change on binding inhibitors

**Figure 10:**
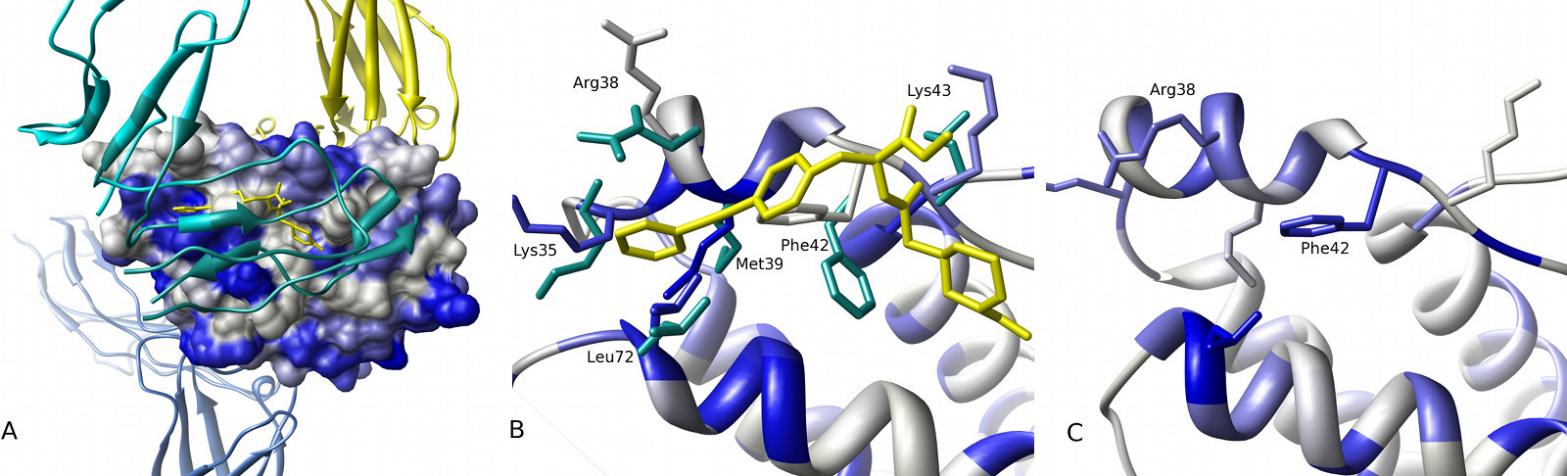
**Residues with variable roles in pocket formation in apo structures may indicate cryptic binding sites**. Analyses of apo structures of IL-2 superfamily members and a set of simulated conformers of apo IL-2 show that residues that have variable roles in pocket formation include those that undergo the significant side-chain conformational rearrangements necessary to accommodate small molecule interface inhibitors. **(A) **Residues that are pocket-lining in a small proportion of the 17 apo homologues of IL-2 are highlighted on the IL-2 surface (dark blue represents the median Provar score of 0.26, with lighter blues indicating a higher or lower score). A region to the left of the α receptor interface (c.f. Figure 9A) is prominent in this visualisation and indicates structural heterogeneity of this region across the superfamily. **(B) **An expanded ribbon view of that part of this region of apo IL-2 coloured as in **(A)**. Side-chains of surface residues in this region are shown in stick representation, superimposed are side chain conformations (cyan) in an inhibitor bound state [PDB:1M48]. The pocket in which the inhibitor binds is not present in the IL-2 crystal structure and large movements of side chains of Arg 38, Lys 35, Met 39, Phe 42 and Leu 72 enable ligand binding. The majority of these residues have pocket forming roles in some structural homologues. **(C) **A ribbon view showing the same region as **(B) **coloured to highlight residues that show variable roles in pocket formation in tCONCOORD simulations of apo IL-2 (dark blue represents the median Provar score of 0.31). The pocket-forming capability of Phe 42 and Arg 38, required for inhibitor binding, is shown by Provar analyses of these IL-2 simulations.

Not all residues forming the IL-2:IL-2R*α *interface show conserved pocket-lining propensity. By colouring the group of residues that are relatively variable in their pocket-lining character (here defined as those pocket-lining residues with scores in the quartiles either side of the median), we obtain Figure 10. In this representation, those residues in equivalent alignment positions across the homologues that show the relatively greatest variability as to whether they form pockets are highlighted in a deep blue (Figure 10A). In this case a region to the left of the *α *interface is notably variable. It has been suggested that when analysing a set of homologues, structural variability in a particular aligned region may imply that the equivalent position on an individual protein in the superfamily is amenable to conformational change [33]. This suggestion seems to be at least partly correct in the case of IL-2. It is known that inhibitors of IL-2:IL-2R association bind at the *α *interface and that formation of their binding pocket requires reorientation of IL-2 side chains precisely in the region highlighted as variable amongst homologues. Figure 10B compares the side chain orientations of residues known to be essential to either receptor or ligand binding between the *α*-receptor-bound and a ligand-bound structure. Of the five residues known to undergo large rearrangements of side chains to accommodate the ligand (Arg 38, Lys 35, Met 39, Phe 42 and Leu 72) [32], three (Lys 35, Met 39 and Leu 72) appear to be variably pocket forming according to Provar analysis of *apo *structures from the superfamily. Of the two residues not highlighted by the analysis of homologues, one (Phe 42) is persistently pocket-lining and the other (Arg 38) has a low propensity to form a pocket. However, these two residues (together with Leu 72) are highlighted as variably pocket-lining in simulated ensemble of conformers of *apo *IL-2 itself (Figure 10C). These results indicate the potential for combining analyses of a superfamily, which can indicate both conserved and variable pocket-lining regions, with simulations of an individual protein, which may provide specific insights into variable regions in the context of a particular interface.

## Discussion

Having a consistent and simple representation of both persistent and variable predicted pockets across arbitrarily large sets of related structures simplifies interpretation of data from which it may otherwise be difficult to extract meaning. Even with a simple example, the predictions of two programs on a single structure, it is not obvious how to quantitatively compare the two pocket predictions. With many conformations the mass of prediction points becomes spread-out and ambiguous as the increasing number of conformers leads to increased local structure variation and smearing due to the changes in the overall global alignments. With the structural dissimilarities that occur in sets of homologues the comparisons can become even harder and necessitate an approach that deals effectively with the problems of structure and sequence alignments. All three of these problems can benefit from the probabilistic approach taken here that assesses the extent to which predictions of pocket-lining atoms or residues hold for the dataset as a whole.

This atom-centred approach avoids the complex issue of how to define each individual pocket and compare these between structures, rather focusing on groups of residues or atoms whose roles in pocket formation are most (or least) variable as a whole. The corollary is that these gross dataset properties do not provide detailed geometric or structural information on individual pockets. The fraction of structures of an ensemble in which equivalent atoms or residues are involved in pocket formation does, of course, not contain all the information present in the original outputs of the prediction programs. For example, if a group of residues form a pocket in all members of an ensemble, their Provar score will readily identify them, but it will not indicate whether the pocket has the same geometry in each structure or not. Our use of persistent and variable to describe pockets reflects the identity of the residues forming the pocket (and indirectly its location) and not necessarily the shape of the pocket. However, in the case of variable pockets, variation in the number and location of residues forming the pocket will almost certainly be accompanied by shape changes. The aim in summarising information using Provar's scoring schemes is to allow insight into large amounts of data that is otherwise difficult to visualise. Once regions of interest are identified then further more focused analyses may be possible.

We have not attempted to re-validate the outputs of existing pocket prediction software (which have in any case been recently critically evaluated [12] for their ability to predict small-molecule binding sites). In this regard, the Provar methodology described here merely aids comparison of different prediction software. Provar analysis readily shows that pocket prediction programs give somewhat different outputs (Figure 1, Tables 1 and 2). Which programs are most suited to particular investigations in the context of analysis of sets of structures remains to be tested, and it may be that it makes sense to combine results of prediction algrorithms that may have different strengths and weaknesses [11]. In this latter case, Provar scores provide a straightforward basis for creating a summary or consensus of several programs (Figure 5).

In the examples that we have presented here, we have mainly been concerned with visualising the persistence/variation of pockets in a protein's conformational or superfamily ensemble in the absence of any ligand, and identifying instances of correlation of pockets formed in these ensembles with persistence or variation of experimentally known ligand binding sites. Such identification (or prediction) of features of ligand binding sites is a widespread application of pocket prediction software when applied to individual structures. The application of the Provar algorithm provides means to visualise the results of analyses on large sets of related structures.

For a kinase superfamily, pockets whose locations are highly conserved across homologues were readily identified and correspond to the enzyme's active and allosteric regulatory sites (Figure 7). There is a potential for similar analysis of other less well understood protein superfamilies to identify common features that would then be the target of functional investigation.

We have seen how Provar visualisations allow us to identify pockets present in members of an ensemble that may be absent from an individual crystal structure. In analysing a conformational ensemble of Bcl-2, Provar analysis indicates an extended binding groove among simulated *apo *conformations compared to that of the crystal structure (Figure 8). We have shown that analyses of conformational ensembles of apo structures usually recover more of known PPI inhibitor binding sites than analyses of single static structures, but that precise outcomes of such analyses are rather dependent on the pocket prediction software used. Again, Provar scoring does provide a convenient approach to comparing such results.

Provar analysis of pocket predictions on simulated ensembles may help guide ligand design efforts by indicating which regions of the proteins surface may adapt to accommodate larger (or smaller) ligands. The residue-based Provar scores themselves could be further analysed to identify subsets of conformations (or subfamilies) in which particular residues are involved in pocket formation. Such subsets may then find a use in computational design efforts, e.g., docking, were they may increase the diversity of candidate ligands, which in turn increases the likelihood of finding one that simultaneously satisfies the requirements of specificity, affinity and ADME-Tox. In the kinases, identifying variable pocket-lining regions bordering conserved regions may be helpful when designing inhibitors that are specific to a particular kinase or kinase subset.

In common with many other forms of structural analyses, the type and quality of inferences made from Provar visualisation depend on an appropriate choice of structure set. We anticipate that a judicious combination of evidence obtained from both sets of homologues (where suitable) and simulated conformational ensembles of individual proteins may provide most insight into variability of pockets, as illustrated with the IL-2:IL-2R interface. In binding-site prediction applications, it is necessary to be careful to exclude any structures that have ligands bound. In the case of comparison of homologous structures, it is necessary to create a set of proteins or domains which are representative of the members of the superfamily, but sufficiently dissimilar from each other to avoid bias to the features of the members with the most numerous structures. However, other applications of the Provar approach may require different criteria, e.g., it may be of interest to compare sets of *apo *and ligand containing structures to identify structural changes leading to pocket formation upon ligand binding that may suggest sites for allosteric regulation.

## Conclusion

The approach to probabilistic analysis of variation of pockets on protein surfaces through mapping the presence or absence of a pocket to the protein atoms and residues that form the pocket, provides a straightforward way of summarising the surface features of many structures. The visualisations of the results of this probability analysis provided useful insight into pocket variability and may find particular application in target characterisation in computational structure-based drug design.

## Methods

### Data sets

All PDB files were downloaded in text format from the RCSB Protein Data Bank [34] and processed to extract only the protein chain of interest. Because artifacts may be generated by pocket prediction programs due to missing residues in the PDB file, care has been taken that these do not overlap with a region of interest.

### Simulations

The structures used in simulation ensemble analysis of proteins involved in protein-protein interactions (Tables 1 and 2) are drawn from the 2P2I database of protein-protein interfaces with known inhibitors [31]. The PDB ids of structures used are listed in Table 4. Prior to simulation, structures were protonated using UCSF Chimera [35] using options to protonate His residues based on their H-bonding pattern. Multiple conformations (250 per run) were generated using tCONCOORD (version 1.0) [20] with the standard input parameter file. For the IL-2 data shown in Figures 2 and 6A simulations were based on an *apo *structure [PDB:1M47] with only the first 50 conformers used in Provar analysis. The atom-based representation (Figure 6A) was obtained by applying Equation 1 and the residue-based ribbon diagram (Figure 6B) using Equation 2. For Bcl-2 (Figure 8) 250 conformers were generated from an *apo *structure[PDB:1GJH] and atom-based Provar scoring (Equation 1) was used to generate Figure 8B. In all cases, binding sites and protein interfaces are defined using those non-hydrogen atoms of the protein within 4.5*Å *of a non-hydrogen atom of the binding partner.

**Table 4:** Structures used in protein-ligand and protein-protein pocket analysis

Target protein (PDB id of *apo *structure)	Inhibitor (PDB id)	Protein partner (PDB id)
Bcl-X*_L_*)	acyl sulfonamide derivative	Bak
(1LXL)	(1YSI)	(1BXL)

MDM2	benzodiazapine derivative	p53
(1Z1M)	(1T4E)	(1YCR)

Xiap apoptosis inhibitor	naphthalenamide derivative	Caspase 9
Bir3 domain (1F9X)	(1TFQ)	(1NW9)

Xiap apoptosis inhibitor	Smac peptidomimetic	SMAC caspase activator
Bir3 domain (1F9X)	(2JK7)	(1G73)

ZipA	indoloquinolizin inhibitor 1	FtsZ
(1F46)	(1S1J)	(1F47)

HPV11 E2 protein	tetrahydrofuran derivative	HPV11 E1 protein
(1RK6)	(1R6N)	(1TUE) (1R6N)

Interleukin 2	diphenyl derivative	IL2-Receptor
(1M47)	(1M48)	(1Z92)

HIV-1 Integrase	chlorophenyl-dihydroquinolin acetic acid	LEDGF
(3L3U)	(3LPT)	(2B4J)

TNFa subunit A	chromen-4-one derivative	TNFa subunit B
(1TNF)	(2AZ5)	(1TNF)

TNF receptor 1a	thiazolidin-4-one derivative	TNF-beta
(1EXT)	(1FT4)	(1TNR)

MDM4 subunit A	chlorobenzyl-phenyl-imidazol derivative	p53
(3DAB)	(3LBJ)	(3DAB)

Structures used in comparative analyses (detailed in Tables 1 and 2) are taken from the 2P2I database of protein-protein complexes with known inhibitors of the protein-protein interaction. In each case, an ensemble is generated from the *apo *structure, pockets from this ensemble are analysed and compared with binding sites/interfaces determined from the inhibitor and protein-protein complex structures. In the case of TNF-alpha, the inhibitor disrupts a protein-protein interface in the native trimer, and the *apo *and protein-protein complex structure files are consequently the same

### Kinase superfamily

We took the single CATH v3.4 [36] representative domains at the S35 level (sequence identity > 35%) for homologues of Phosphorylase Kinase domain 1 (CATH superfamily ID: 3.30.200.20), giving 93 protein chains. Structures were downloaded from the PDB and processed as outlined above. Pocket predictions using PASS (for the 91 structures that gave valid output) were used to generate residue-based probabilities using Provar scoring with Equation 3 and multiple sequence alignments from ClustalW2 [37].

### Homologues of IL-2

Seventeen *apo *structures were identified among the representative S35 domains in CATH homologous superfamily 1.20.1250.10. An overall sequence alignment was generated using MUSTANG [38] using only the observed amino acid sequences found in the PDB files. In order to map Provar results for Figures 9, 10A and 10B onto a receptor-bound structure, the receptor-bound IL-2 chain [PDB:2B5I] was included in the MUSTANG alignments, but ignored during Provar calculations. Equation 4 was used for scoring in Figures 9 and 10. Pocket predictions were made for each structure with LIGSITE^-cs ^(shown in Figure 9), PASS and fpocket and averaged using Provar. MDPocket was also used to map fpocket-based scores to residue for this dataset. Figure 10C used 250 conformers from a tCONCOORD simulation of *apo *IL2 [PDB:1M47] to define probabilities. To enable comparison between scores generated from different datasets and methods, the subsets for analysis and visualization are selected using the quartiles of each distribution of non-zero probability scores, where residues which are relatively persistently pocket forming have scores (*Q3*) (i.e. in the top 25%) and those which are relatively variable fall in the range *(Q3-Q1) *in each case.

### Pocket prediction

Sets of related structures (conformers, homologs etc.) were processed as a batch with either PASS (v2.0.36) or LIGSITE^-cs ^or fpocket v1.0. PASS was run with the'-more' flag and only the files ending'probes_r.pdb', comprising the final layer of individual probe spheres were used in the Provar analysis. fpocket was run using default'SET1' parameters. LIGSITE^-cs ^was run with default parameters, with the exception of a grid size of 0.5*Å*, and files ending'pocket-r.pdb', containing the centroids of all pocket-prediction spheres were used in analysis. In all cases, atoms were defined as pocket-lining by Provar if within 3.75*Å *of any predicted pocket position (centres of probe spheres). Provar scores are potentially sensitive to the precise placement of pocket prediction spheres with respect to the protein atoms. Too coarse a representation of pockets by the pocket prediction software can lead to artifacts in mapping to atoms. However, comparative tests on tCONCOORD generated ensembles show that use of this distance criterion in conjunction with any pocket prediction sphere radius or grid spacing ≤ 1.0*Å *gives consistent scores (e.g. the Matthews correlation coefficients of atom-based scores for LIGSITE^-cs ^between grid size = 0.5*Å *and all grid sizes ≥ 0.2*Å *and ≤ 1.0*Å *are > 0.88).

### Software implementation

The Provar method is implemented as a series of MATLAB modules driven from a single user-modifiable configuration file (describing paths and input data types). Presently, pocket descriptions can be read from fpocket, LIGSITE^-cs^, SiteMap and PASS format output files. A PDB input format is also supported for those programs, such as CASTp, which directly output atom or residue based scores in this format. Modules were developed and run in MATLAB 7.4.0.287 (R2007a) running on OS X (Leopard). PDB files manipulated via BioJava v1.7 [39] modules. PASS (v2.0.36), LIGSITE^-cs ^and fpocket were run on an IBM 3550 Dual Xeon X5355 @ 2.66GHz CPU, 16Gb Memory, SuSE Linux workstation. tCONCOORD (v1.0) was run on nodes of a Rocks Cluster. All structures were visualised and rendered using UCSF Chimera (v1.5.2) [35].

## Availability and requirements

The Matlab modules for Provar, together with a description of their use and example datasets, are freely available at http://people.cryst.bbk.ac.uk/~ubcg66a/software.html. In addition to the base implementation of Matlab (version 7.4 or later), the Statistics Toolbox is required.

## Competing interests

The authors declare that they have no competing interests.

## Authors' contributions

PA carried out all programming. PA and AP carried out data analysis. All authors contributed to design and development of the study. PA, DSM, IN and MAW wrote the paper. All authors read and approved the final manuscript.
